# Supplementary figures and images for: General and specific stress mindsets: Links with college student health and academic performance
Source: PLoS One. 2021 Sep 8;16(9):e0256351. doi: 10.1371/journal.pone.0256351 (PMC8425538; doi:10.1371/journal.pone.0256351)

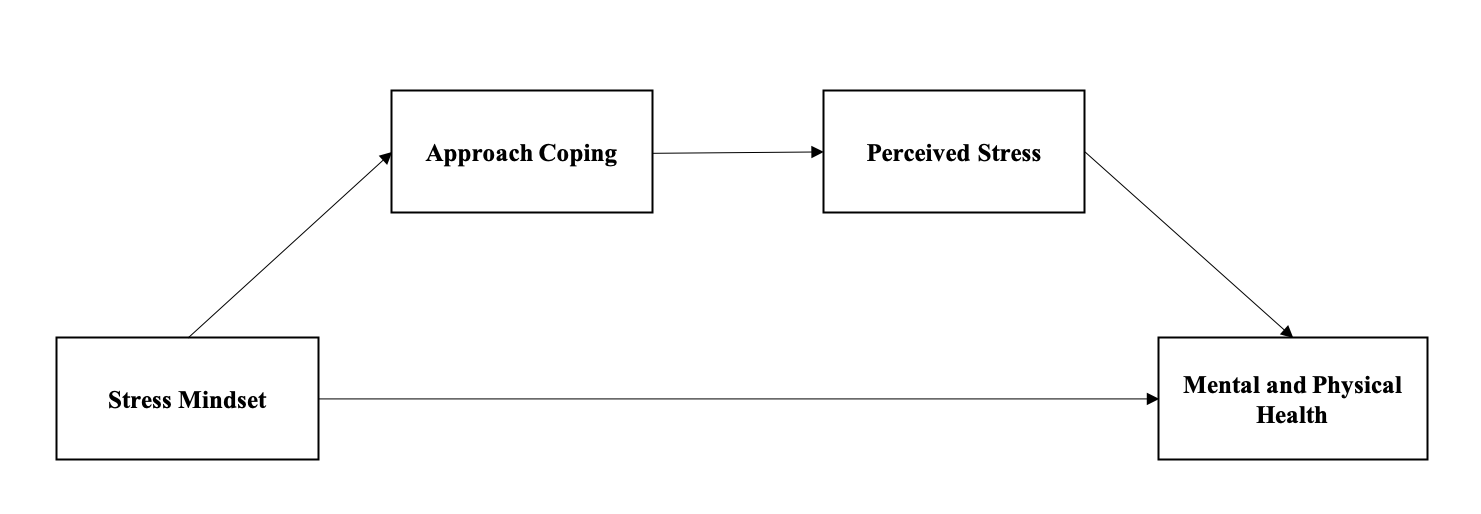

Supplement: S1 Fig — (TIF) [file pone.0256351.s001.tif]
