# Supplementary material for: General and specific stress mindsets: Links with college student health and academic performance
Source: PLoS One. 2021 Sep 8;16(9):e0256351. doi: 10.1371/journal.pone.0256351 (PMC8425538; doi:10.1371/journal.pone.0256351)
Supplement: S1 Table — Results are from four multivariate analyses of variance examining differences in study variables as a function of gender and sample (for group differences by sample see S2 Table). Positive effect sizes indicate that the men were higher on a particular variable, negative effect sizes indicate that the women sample was higher on a particular variable. (PDF) [file pone.0256351.s002.pdf]

S1 Table. Gender Differences in Stress Mindsets, Perceived Stress, Stressful Life Events, Coping, and Mental and Physical Health

	Wilks' λ	Men	Women	*F*	*d*
		Mean (*SD*)			
Stress Mindsets	.956			*(5,476)* 4.43**	
General		2.02 (.82)	1.83 (.81)	8.41**	.24
Acute Controllable		2.34 (.86)	2.39 (.82)	.08	-.06
Chronic Controllable		2.54 (.82)	2.35 (.90)	7.37**	.22
Acute Uncontrollable		1.38 (.88)	1.40 (.76)	.08	-.03
Chronic Uncontrollable		1.85 (1.05)	1.53 (.99)	12.28***	.32
Stress	.977			*(2,482)* 5.78**	
Perceived Stress		18.93 (7.32)	20.95 (7.40)	11.56**	-.28
Stressful Life Events		549.84 (320.18)	558.86 (303.19)	1.53	-.03
Coping	.962			*(4,482)* 4.71**	
Approach Coping		3.80 (.67)	3.75 (.65)	.75	.08
Social Coping		3.01 (.93)	3.32 (.93)	11.21**	-.33
Distractive Coping		2.79 (.72)	2.88 (.77)	2.35	-.11
Avoidant Coping		2.21 (.73)	2.32 (.82)	8.97**	-.26
Health	.956			*(3, 426)* 6.52	
Mental Health Symptoms		1.07 (.72)	1.38 (.85)	18.02***	-.39
Self-Reported Poor Health		2.44 (1.01)	2.64 (.98)	5.87*	-.21
Number of Days Health Interfered		5.33 (5.86)	7.02 (6.69)	9.84**	-.27

Note. Results are from four multivariate analyses of variance examining differences in study variables as a function of gender and sample (for group differences by sample see S2 Table). Positive effect sizes indicate that the men were higher on a particular variable, negative effect sizes indicate that the women sample was higher on a particular variable.

$^{*}p < .05$, $^{**}p < .01$, $^{***}p < .001$.
