# Supplementary material for: General and specific stress mindsets: Links with college student health and academic performance
Source: PLoS One. 2021 Sep 8;16(9):e0256351. doi: 10.1371/journal.pone.0256351 (PMC8425538; doi:10.1371/journal.pone.0256351)
Supplement: S2 Table — Results are from four multivariate analyses of variance examining differences in study variables as a function of gender and sample (for group differences by gender see S1 Table). Positive effect sizes indicate that the participant pool sample was higher on a particular variable, negative effect sizes indicate that the MTurk sample was higher on a particular variable. (PDF) [file pone.0256351.s003.pdf]

S2 Table: Sample Differences in Mindsets, Perceived Stress, Stressful Life Events, Coping, and Mental and Physical Health

	Wilks' λ	Participant Pool Sample	MTurk Sample	F	d
		Mean (*SD*)			
Mindsets	.836			*(5,476)* 18.74***	
General		2.10 (.75)	1.60 (.82)	47.01***	.64
Acute Controllable		2.42 (.78)	2.30 (.90)	1.36	.14
Chronic Controllable		2.46 (.82)	2.38 (.94)	.34	.09
Acute Uncontrollable		1.24 (.71)	1.61 (.89)	28.26***	-.48
Chronic Uncontrollable		1.68 (1.01)	1.61 (1.05)	.51	.06
Stress Measures	.816			*(2,482)* 54.47***	
Perceived Stress		19.50 (7.15)	21.28 (7.72)	5.18*	-.24
Stressful Life Events		445.76 (213.92)	727.02 (354.84)	108.92***	-1.01
Coping	.982			*(4, 482)* 2.16*	
Approach Coping		3.76 (.61)	3.79 (.73)	.26	-.04
Social Coping		3.27 (.90)	3.09 (1.00)	3.14*	.19
Distractive Coping		2.86 (.73)	2.86 (.73)	1.03	.00
Avoidant Coping		2.21 (1.78)	2.29 (.82)	.98	-.10
Health	.972			*(3, 426)* 4.05	
Mental Health Composite		1.16 (.73)	1.41 (.91)	8.57**	-.31
Self-Reported Health		2.43 (.97)	2.74 (.10)	8.30**	-.32
Number of Days Health Interfered		5.66 (5.89)	7.33 (7.20)	4.12*	-.27

Note. Results are from four multivariate analyses of variance examining differences in study variables as a function of gender and sample (for group differences by gender see S1 Table). Positive effect sizes indicate that the participant pool sample was higher on a particular variable, negative effect sizes indicate that the MTurk sample was higher on a particular variable.

* $p < .05$, ** $p < .01$, *** $p < .001$.
