# Supplementary material for: General and specific stress mindsets: Links with college student health and academic performance
Source: PLoS One. 2021 Sep 8;16(9):e0256351. doi: 10.1371/journal.pone.0256351 (PMC8425538; doi:10.1371/journal.pone.0256351)
Supplement: S5 Table — (PDF) [file pone.0256351.s006.pdf]

S5 Table. Indirect Effects of Specific Stress Mindset (Chronic Controllable) on Health through Approach Coping and Perceived Stress

	Mental Health Symptoms		General Self-Reported Poor Health		Number of Days Health Interfered with Normal Activity	
	Point Estimate (95% CI)	R^2	Point Estimate (95% CI)	R^2	Point Estimate (95% CI)	R^2
Indirect Effects (IE)						
Specific IE through Approach Coping	-.008 (-.023, .006)	—	-.013 (-.035, .006)	—	.029 (-.125, .198)	—
Specific IE through Perceived Stress	-.105 (-.158, -.054)	—	-.091 (-.140, -.045)	—	-.556 (-.866, -.279)	—
Specific IE through Approach Coping and Perceived Stress	-.016 (-.033, -.004)	—	-.014 (-.029, -.003)	—	-.085 (-.175, -.017)	—
Total IE	-.129 (-.186, -.075)	.565	-.118 (-.174, -.068)	.309	-.612 (-.926, -.320)	.266

Note. Confidence intervals are 95% bias-corrected bootstrap confidence intervals with 10,000 resamples calculated using Hayes's (2018) PROCESS macro for SPSS (v. 26.0); indirect effects are significantly different from zero when the associated confidence interval does not contain zero. Point estimates are from serial mediation models including mindset as the predictor, approach coping and perceived stress as serial mediators (in that order), and the health measures as outcomes (see S1 Figure); all analyses are controlling for sample, gender, and history of stressful life events; R^2 values are for full models including all predictors and control variables.
