# Supplementary material for: General and specific stress mindsets: Links with college student health and academic performance
Source: PLoS One. 2021 Sep 8;16(9):e0256351. doi: 10.1371/journal.pone.0256351 (PMC8425538; doi:10.1371/journal.pone.0256351)
Supplement: S1 Appendix — (DOCX) [file pone.0256351.s009.docx]

## S1 Appendix

## Adherence to Preregistered Analysis Plan

The analysis plan for was preregistered at: <https://osf.io/c97ng/?view_only=0162820d6c6e4086937b90afb97fe10d>.

As indicated in the analysis plan, some data for the study had already been collected at the time that the study was preregistered. Specifically, we had already recruited 449 out of the final 498 participants at the time of preregistration. However, none of the data were analyzed or even organized for analysis at the time of preregistration.

All preregistered analyses were conducted, and most are included in the main manuscript. In the interest of creating a briefer, more readable manuscript, we offer one of these preregistered analyses in the supporting information rather than the main manuscript. Specifically, we preregistered that we would examine whether a history of stressful life events explains the relationship between stress mindset and health. Research has consistently shown that higher levels of stress are predictive of poorer health (5), and research on stress mindsets shows that people who have experienced more stressful life events tend to endorse a more stress-is-debilitating mindset (4). What is unclear from previous research is whether the link between stress mindsets and health may be partially explained by the fact that people who view stress as more debilitating also have had more exposure to stress. Thus, an additional goal of this study was to examine whether the link between stress mindsets and health remains once a history of stressful life events is taken into account. We report the results of these analyses in the S2 Appendix.

Based on the suggestions of an anonymous reviewer, we performed a confirmatory factor analysis to examine the factor structure of the adapted, 3-item General Stress Mindset Scale and newly developed Source-specific Stress Mindset Scales. This analysis was not part of the preregistration and is referenced in the main manuscript and reported in more detail in S3 Appendix.
