# Supplementary material for: General and specific stress mindsets: Links with college student health and academic performance
Source: PLoS One. 2021 Sep 8;16(9):e0256351. doi: 10.1371/journal.pone.0256351 (PMC8425538; doi:10.1371/journal.pone.0256351)
Supplement: S2 Appendix — Supplemental preregistered analysis examining whether stress mindsets are associated with health, controlling for history of stressful life events. (DOCX) [file pone.0256351.s010.docx]

## S2 Appendix

## Supplemental preregistered analysis: History of stressful life events as a potential explanatory variable in the link between stress mindsets and health

An important gap in the literature is in the link between previous experience of stress, stress mindsets, and health. Research has consistently shown that higher levels of stress are predictive of poorer health (5), and research on stress mindsets shows that people who have experienced more stressful life events tend to endorse a more stress-is-debilitating mindset (4). What is unclear from previous research is whether the link between stress mindsets and health may be partially explained by the fact that people who view stress as more debilitating also have had more exposure to stress. A preregistered goal of this study was to examine whether the link between stress mindsets and health remains once a history of stressful life events is taken into account.

To address the question of whether history of life events may serve as a potential explanatory variable in the link between mindsets and health we first examined correlations among history of stressful life events, stress mindsets, and health. Next, hierarchical multiple regression analyses were conducted to examine whether there would be a significant association between mindsets and health once history of stressful life events was taken into account.

Consistent with previous research, participants who had experienced more lifetime stress also reported poorer mental and physical health, even controlling for sample and gender (see Table A). We did not, however, find significant associations between any of the stress mindset measures and history of stressful life events once variance associated with sample and gender was partialled out, suggesting that stressful life events were unlikely to act as an explanatory variable in the link between stress mindsets and health.

Still, to address our preregistered question of whether the link between stress mindsets and health remains once history of stressful life events is taken into account, a series of hierarchical multiple regression analyses was conducted; one predicting mental health symptoms, one predicting general self-reported poor health, and one predicting the number of days health interfered with normal activities. In each analysis: (a) gender and sample were entered as control variables in the first step of the model, (b) the stressful life events weighted sum score was entered in the second step of the model, and (c) the set of five stress mindset variables (general stress mindsets plus each of the four source-specific stress mindsets) was entered in the third step of the model. The five stress mindsets variables are only moderately correlated with one another (*r*s ranging from .01 to .33), and multicollinearity diagnostics indicated no significant problems with multicollinearity, so the five mindsets variables were considered simultaneously in a single step in these models. Results of these analyses are presented in Table A.

Across the three models, stress mindsets as a group did predict health over and above the prediction of stressful life events, suggesting that history of stressful life events is not an explanatory variable in the link between stress mindsets and health. Stress mindset in the chronic, controllable stressful situation (having a quiz every class for which you feel you can adequately prepare) was the most pervasively related to health, such that an enhancing mindset was associated with better mental and physical health, controlling for history of stressful life events and the other mindsets. Stress mindset in the acute, controllable and acute, uncontrollable stressful situations were also significantly associated with health, but less pervasively so. A more enhancing mindset in the acute, controllable situation was related to lower mental health symptoms and marginally fewer days in which health interfered with normal activities, but not general self-reported health. A more enhancing mindset in the acute, uncontrollable situation was related to better overall perceived health, and lower mental health symptoms. Interestingly, general stress mindset was not uniquely associated with any of the health outcomes once the source-specific mindsets were taken into account. Across the three models, stress mindsets as a group accounted for between 3.9% and 7.7% of the variance in health measures.

Table A. Hierarchical Multiple Regression Analyses Predicting Health Measures from History of Stressful Life Events and Stress Mindset

|  |  | Mental Health Symptoms | | | | |  | General Health | | | | |  | Number of Days Health Interfered with Activities | | | | |
| --- | --- | --- | --- | --- | --- | --- | --- | --- | --- | --- | --- | --- | --- | --- | --- | --- | --- | --- |
| Predictors |  | Δ*R*^2^ |  | *b* (*se*) |  | *sr*^2^ |  | Δ*R*^2^ |  | *b* (*se*) |  | *sr*^2^ |  | Δ*R*^2^ |  | *b* (*se*) |  | *sr*^2^ |
|  |  |  |  |  |  |  |  |  |  |  |  |  |  |  |  |  |  |  |
| **Step 1** |  | **.074^***^** |  |  |  |  |  | **.055^***^** |  |  |  |  |  | **.033^**^** |  |  |  |  |
| Control variables^a^ |  |  |  |  |  |  |  |  |  |  |  |  |  |  |  |  |  |  |
| **Step 2** |  | **.109^***^** |  |  |  |  |  | **.062^***^** |  |  |  |  |  | **.067^***^** |  |  |  |  |
| Stressful life events (weighted) |  |  |  | .001 (.000)^***^ |  | .108 |  |  |  | .001 (.000)^***^ |  | .062 |  |  |  | .006 (.001) ^***^ |  | .067 |
| **Step 3: Mindsets** |  | **.077^***^** |  |  |  |  |  | **.039^**^** |  |  |  |  |  | **.040^**^** |  |  |  |  |
| General |  |  |  | -.07 (.05) |  | .003 |  |  |  | -.05 (.06) |  | .001 |  |  |  | -.06 (.44) |  | .000 |
| Acute Controllable |  |  |  | -.09 (.04)^*^ |  | .007 |  |  |  | -.02 (.06) |  | .000 |  |  |  | -.41 (.39) |  | .002 |
| Chronic Controllable |  |  |  | -.12 (.04)^**^ |  | .015 |  |  |  | -.12 (.05)^*^ |  | .009 |  |  |  | -1.25 (.36) ^**^ |  | .025 |
| Acute Uncontrollable |  |  |  | -.15 (.04)^***^ |  | .020 |  |  |  | -.16 (.06) ^**^ |  | .015 |  |  |  | -.16 (.38) |  | .000 |
| Chronic Uncontrollable |  |  |  | .00 (.03) |  | .000 |  |  |  | -.01 (.04) |  | .000 |  |  |  | .44 (.30) |  | .004 |
| Adjusted *R*^2^ |  | .248^***^ | | | | |  | .141^***^ | | | | |  | .123^***^ | | | | |
| Each column represents a separate hierarchical multiple regression analysis, with the dependent variable listed in the column header. ^a^ control variables are gender (0 = *men,* 1 = *women*) and sample (0 = *psychology participant pool*, 1 = *Amazon Mechanical Turk*). *sr*^2^ = squared semi-partial correlation.  ^*^ *p* < .05, ^**^ *p* < .01, ^***^ *p* < .001. | | | | | | | | | | | | | | | | | | |
