# Supplementary material for: General and specific stress mindsets: Links with college student health and academic performance
Source: PLoS One. 2021 Sep 8;16(9):e0256351. doi: 10.1371/journal.pone.0256351 (PMC8425538; doi:10.1371/journal.pone.0256351)
Supplement: S3 Appendix — (DOCX) [file pone.0256351.s011.docx]

## S3 Appendix

## Confirmatory factor analysis to examine factor structure of the adapted Stress Mindset Scale measures

To examine the factor structure of the adapted 3-item General Stress Mindset Scale and the newly developed Source-specific Stress Mindsets Scales, a confirmatory factor analysis was conducted to evaluate the fit of a baseline measurement model with general and stressor-specific mindsets items loading onto five correlated first-order factors (see Table A for items and factor loadings, see Figure A for path diagram of the measurement model). Note that this analysis was suggested by a reviewer and therefore was not part of the preregistration. To account for possible shared variance among reverse-worded items across factors that could affect model fit, we took a correlated traits-correlated uniqueness approach and specified correlated error terms among the reverse-worded items (see Brown, 2003; Marsh, 1996). Models were estimated in Mplus version 8.6 (Muthén & Muthén, 2017) using full information maximum likelihood estimation, and omnibus model fit was evaluated with regard to the root mean square error of approximation (RMSEA, Steiger & Lind, 1980; values .05 to .08 indicate adequate model fit, ≤ .05 indicate good model fit, Browne & Cudeck, 1993), the comparative fit index (CFI, Bentler, 1990; values from .90 to .95 and higher indicate good model fit, Hu & Bentler, 1999), and the standardized root mean square residual (SRMR, values ≤ .08 indicate good model fit, Hu & Bentler, 1999). The model χ^2^ statistic is also reported, but not used to evaluate model fit because it often rejects models that are a good fit to the data (Bentler & Yuan, 1999). Fit statistics, with the exception of the SRMR statistic which exceeds the suggested cutoff, indicated that the hypothesized model presented an adequate fit to the data χ^2^ (75) = 205.49, *p* < .001; RMSEA = .059 [90% CI .050–.069]; CFI = .948; SRMR = .113.

**Table A. Items and standardized factor loadings from stress mindsets measurement model**

|  | Standardized Factor Loading | Standard Error |
| --- | --- | --- |
| **General Stress Mindset (SMG)** |  |  |
| Experiencing stress facilitates my learning and growth (smg1) | .815 | .015 |
| Experiencing stress debilitates my performance and productivity (smg2) | -.577 | .040 |
| The effects of stress are positive and should be utilized (smg3) | .761 | .033 |
|  |  |  |
| **Source-Specific Stress Mindset – Acute Controllable (SMS-AC)** |  |  |
| Experiencing this kind of stress facilitates my learning and growth (smsac1) | .889 | .015 |
| Experiencing this kind of stress debilitates my performance and productivity (smsac2) | -.571 | .037 |
| The effects of this kind of stress are positive and should be utilized (smsac3) | .852 | .026 |
|  |  |  |
| **Source-Specific Stress Mindset – Chronic Controllable (SMS-CC)** |  |  |
| Experiencing this kind of stress facilitates my learning and growth (smscc1) | .848 | .013 |
| Experiencing this kind of stress debilitates my performance and productivity (smscc2) | -.620 | .032 |
| The effects of this kind of stress are positive and should be utilized (smscc3) | .914 | .020 |
|  |  |  |
| **Source-Specific Stress Mindset – Acute Uncontrollable (SMS-AU)** |  |  |
| Experiencing this kind of stress facilitates my learning and growth (smsau1) | .922 | .028 |
| Experiencing this kind of stress debilitates my performance and productivity (smsau2) | -.436 | .051 |
| The effects of this kind of stress are positive and should be utilized (smsau3) | .808 | .043 |
|  |  |  |
| **Source-Specific Stress Mindset – Chronic Uncontrollable (SMS-CU)** |  |  |
| Experiencing this kind of stress facilitates my learning and growth (smscu1) | .831 | .015 |
| Experiencing this kind of stress debilitates my performance and productivity (smscu2) | -.625 | .029 |
| The effects of this kind of stress are positive and should be utilized (smscu3) | .881 | .023 |
| *Note*. For source-specific stress mindset measures, participants respond to each item in reference to the sources of stress presented in Table 1 in the main manuscript. Estimates (fully standardized factor loadings) are drawn from the measurement model presented in Fig 1 in the main manuscript; all factor loadings are significantly different from zero at *p* < .001. | | |


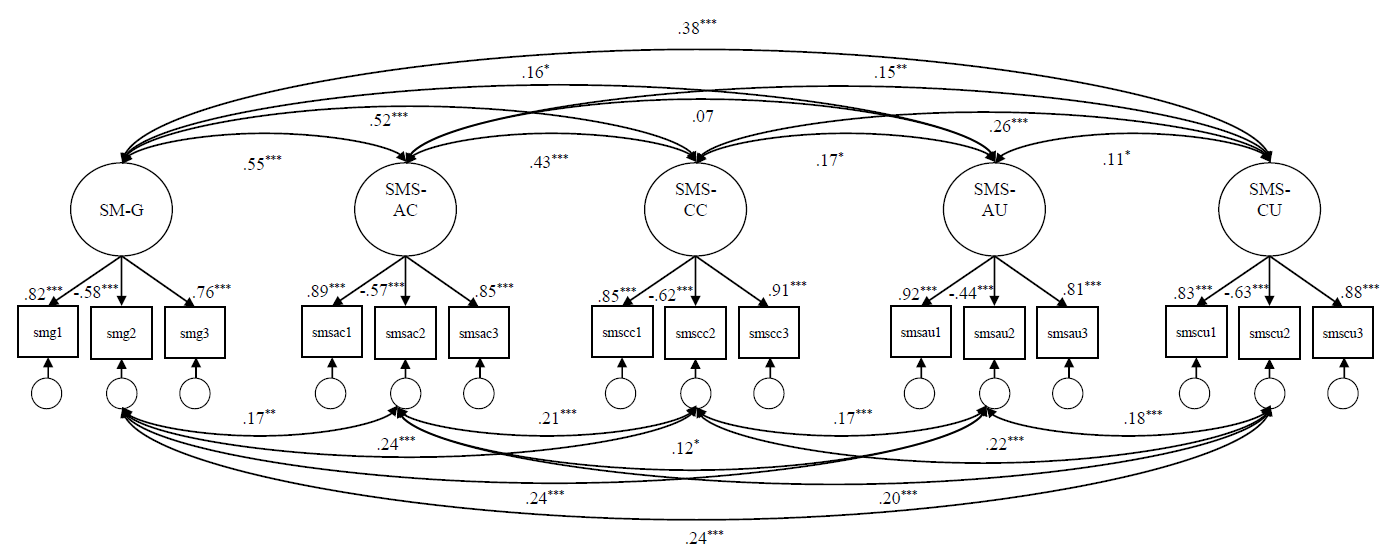


**Fig A. Path diagram and parameter estimates for stress mindsets measurement model**

*Note.* Abbreviations are as follows: SM-G=*General Stress Mindset*, SMS-AC=*Source-Specific Mindset, Acute Controllable*, SMS-CC= *Source-Specific Mindset, Chronic Controllable*, SMS-AU= *Source-Specific Mindset, Acute Uncontrollable*, SMS-CU= *Source-Specific Mindset, Chronic Uncontrollable*. Observed variables are represented with squares/rectangles and latent/unobserved variables are represented with circles/ellipses. Observed variables in the model are responses to individual stress mindset items, and latent variables are hypothesized general and source-specific stress mindsets. Disturbance terms have been omitted from the path diagram for clarity of presentation, including disturbance terms. Curved arrows below the observed variables represent an estimated methods correlation for reverse-worded items; curved arrows above the latent variables indicate correlations among latent variables. ^*^ *p* < .05, ^**^ *p* < .01, ^***^ *p* < .001.

**References**

Bentler, P. M. (1990). Comparative fit indexes in structural models. *Psychological Bulletin, 107*(2), 238–246. https://doi.org/10.1037/0033-2909.107.2.238

Bentler, P.M., & Yuan, K..H. (1999). Structural equation modeling with small samples: Test statistics. *Multivariate Behavioral Research, 34*(2), 181–197. https://doi.org/10.1207/S15327906Mb340203

Brown, T. (2003). Confirmatory factor analysis of the Penn State Worry Questionnaire: Multiple factors or method effects? *Behaviour Research and Therapy,* *41*(12), 1411–1426. https://doi.org/10.1016/S0005-7967(03)00059-7

Browne, M., & Cudeck, R. (1993). Alternative ways of assessing model fit. In K. A. Bollen & J. S. Long (Eds.), *Testing structural equation models* (pp. 136–159). Newbury Park, CA: Sage.

Corwyn, R. F. (2000). The factor structure of global self-esteem among adolescents and adults. *Journal of Research in Personality, 34*(4), 357–379. https://doi.org/10.1006/jrpe.2000.2291

Hu, L.-T., & Bentler P. M. (1999). Cutoff criteria for fit indices in covariance structure analysis: Conventional criteria versus new alternatives. *Structural Equation Modeling, 6*(1), 1–55. https://doi.org/10.1080/10705519909540118

Marsh, H. W. (1996). Positive and negative global self-esteem: A substantively meaningful distinction or artifactors? *Journal of Personality and Social Psychology,* *70*(4), 810–819. https://doi.org/10.1037//0022-3514.70.4.810

Muthén, L.K., & Muthén, B.O. (1998-2017). *Mplus User’s Guide*. Eighth Edition. Los Angeles, CA: Muthén & Muthén.

Steiger, J. H., & Lind, J. C. (1980, May). *Statistically based tests for the number of common factors*. Paper presented at the Annual Meeting of the Psychometric Society, Iowa City, IA.
